# Repeated cocaine reorganizes striatal ensembles along the ventral–dorsal axis

**DOI:** 10.64898/2026.09.15.751852

**Published:** 2026-09-22

**Authors:** Michael Z. Leonard, Hannah B. Reiley, Ainoa Konomi Pilkati, Kathryn J. Bjornson, Maxime Chevée, Erin S. Calipari

## Abstract

Drugs of abuse produce lasting adaptations in the striatum. A prominent theory holds that the early reinforcing effects of cocaine preferentially engage the ventral striatum, and repeated cocaine use recruits dorsal striatal systems associated with habitual drug-related behavior. However, this transition has largely been conceptualized on the population level, obscuring how it is implemented within individual neurons and how repeated drug exposure reorganizes drug responsivity within these circuits. Here, we used single-cell calcium imaging to examine ventral and dorsal striatal responses to acute and repeated cocaine exposure in mice. Cocaine broadly suppressed neuronal activity throughout the ventral and dorsal striatum. However, repeated exposure produced opposing changes in cocaine-modulated neurons across the ventral–dorsal axis. The cocaine-activated population was reduced ventrally while expanding dorsally. Unique to dorsal striatum, repeated cocaine engendered two distinct features of activity: 1) Cocaine-activated cells had weak baseline coupling with the surrounding neuronal population and 2) The emergence of a subpopulation of neurons exhibiting regular, slow rhythmic activity in response to cocaine. Thus, repeated cocaine effects reflect a change in how the circuit is organized rather than simply how strongly it is engaged. This organization may allow cocaine to engage patterns of dorsal striatal activity that are largely absent under baseline conditions, creating a circuit state that becomes increasingly specific to the presence of the drug.

## Full Text Availability

The license terms selected by the author(s) for this preprint version do not permit archiving in PMC. The full text is available from the preprint server.

